# 

**Published:** 1841-10

**Authors:** 


### WESTERN JOURNAL

OF

### MEDICINE AND SURGERY.

### COLLABORATORS.

EDWARD H. BARTON, M. D. *Professor of the Theory and Practice of Medicine, in the Medical College of Louisiana.*

WILLIAM J. BARBEE, M. D. *Cincinnati, Ohio.*

GEORGE W. BAYLESS, M. D. *Demonstrator of Anatomy in the Louisville Medical Institute.*

SOLON BORLAND, M. D., *Memphis, Tennessee.*

A. H. BUCHANAN, M. D. *Columbia, Tennessee.*

CHARLES CALDWELL, M. D. *Professor of the Institutes of Medicine and Medical Jurisprudence in the Louisville Medical Institute.*

SAMUEL A. CARTWRIGHT, M. D. *Mississippi.*

A. CLAPP, M. D. *New Albany, Indiana.*

JEDEDIAH COBB, M. D. *Professor of Anatomy in the Louisville Medical Insti tu*

THOMAS W. COLESCOTT, M. D. *Louisville, Ky.*

JOHN ESTEN COOKE, M. D. *Professor of the Theory and Practice of Medicine in the Louisville Medical Institute.*

SAMUEL D. GROSS, M. D. *Professor of Surgery in the Louisville Medical Institute.*

JOHN HARDIN, M. D. *Munfordsville, Ky.*

JOHN P. HARRISON, M. D. *late Professor of Materia Medica, in the Cincinnati College.*

JOHN F. HENRY, M. D. *Bloomington Illinois*

SAMUEL P. HILDRETH, M. D. *Marietta, Ohio.*

SAMUEL HOGG, M. D. *Nashville, Tennessee.*

M. Z. KREIDER, M. D., *Lancaster, Ohio.*

MOSES L. LINTON, M. D. *Springfield, Kentucky.*

HENRY MILLER, M. D. *Professor of Obstetrics and the Diseases of Women and Children in the Louisville Medical Institute.*

JOHN W. MONETTE, M. D. *Washington, Mississsippi.*

SAMUEL B. RICHARDSON, M. D., *Lecturer on Anatomy and Surgery, &c, Louisville, Ky.*

JOHN L. RIDDELL, M. D. *Professor of Chemistry in the Medical College of Louisiana.*

LANDON C. RIVES, M. D. *late Professor of Obstetrics in the Medical Department of the Cincinnati College.*

CHARLES W. SHORT, M. D. *Professor of Materia Medica in the Louisville Medical Institute.*

G. TROOST, M. D. *Professor of Chemistry and Mineralogy in the University of Nashville.*

AMASA L. TROWBRIDGE, M. D. *Professor of Surgery, Willoughby University, Ohio.*

JOHN A. WARDER, M. D. *Cincinnati, Ohio.*

WILLIAM WOOD, M. D. *Cincinnati, Ohio*

a scientific and settled character; and the results of their enquiries be made available, in practice, to the whole medical profession. D.

---

BIBLIOGRAPHICAL NOTICE.

A System of Practical Medicine, comprised in a series of original dissertations. Arranged and edited by Alexander Tweedie, M. D. F. R. S. &c. With notes and additions, by W. W. Gerhard, M. D. &c. Philadelphia. Lea & Blanchard. 1841.

We have just received a copy of this work, completed as it is in five volumes. We of course could not, in any reasonable limits, give even a very condensed view of the monographs of which it is made up; and, not having called their attention to the volumes as they came out, we would now earnestly recommend to our country brethren to supply themselves with this ready and valuable book of reference. The contents of the volumes are as follows:

Vol. 1. Dissertations on Fevers, General Pathology, Inflammation, and Diseases of the Skin. By Drs. Symonds, Allison, Christison, Schedel, Locock, Gregory, Burrows, and Shapter.

Vol. 2. Dissertations on Nervous Diseases. By Drs. Hope, Prichard, Bennett, Taylor, and Thompson.

Vol. 3. Dissertations on Diseases of the Organs of Respiration. By Drs. Williams, Carpenter, and Joy.

Vol. 4. Dissertations on Diseases of the Digestive, Urinary, and Uterine Organs. By Drs. Joy, Symonds, George Budd, Thompson, Christison, Ferguson, and Simpson.

Vol. 5. Dissertations on Hæmorrhages, Dropsy, Rheumatism, Gout, Scrofula, etc., etc., etc., with a Formulary and General Index. By Drs. Burrows, George Budd, Watson, Shapter, Rowland, William Budd, Farre, and Joy.

*Receipts for the Medical Journal for the month of September,* 1841.

| | |
|---|---|
| Dr. J. Rubey, Abington, Ia. | $5 00 |
| B. S. Shelburn, Taylorsville, Ky. | 10 00 |
| B. Yandell, Benton, Miss. | 10 00 |
| H. J. Holmes, Spring Ridge, Miss. | 5 00 |
| J. C. Harris, Jefferson, Ala. | 5 00 |
| F. W. Campbell, Spring Hill, Ark. | 15 00 |
| O. H. P. Stone, Lexington, Mo. | 10 00 |
| J. Andrews, Rodney, Miss. | 8 00 |
| N. G. Sales, Cranesville, O. | 5 00 |
| A. Sears, Meredocia, Ill. | 5 00 |
| Thos. H. Todd, Starkville, Ala | 3 00 |
| S. G. Maus, Brownsville, Ill | 5 00 |
| D. Morgan, Evansville, Ia. | 5 00 |
| J. Lyon, Vienna, Ala. | 10 00 |
| Drs. Denny & Jones, Suggsville, Ala | 5 00 |
| Dr. J. G. Scull, Black Bluff, Ala. | 7 00 |
| J. C. Spottswood, Athens, Ala. | 10 00 |

The Western Journal of Medicine and Surgery is published monthly by the undersigned, at the corner of Main and Fifth streets, Louisville, at $5 per annum, payable in advance. Each number contains from 80 to 84 pages making two volumes in the year of about 500 pages.

Contributions to its pages by the Physicians of the Valley of the Mississippi, addressed to the Editors, are respectfully solicited.

Letters on business, to be addressed, postage paid, to the publishers.

☞Postmasters, by the regulations of the Postoffice Department, will frank letters containing subscription money, and all remittances so franked are at the risk of the publishers.

*July* 25, 1841. PRENTICE & WEISSINGER.

